# Correction: Palladium-catalysed 5-*endo-trig* allylic (hetero)arylation

**DOI:** 10.1039/d0sc90169e

**Published:** 2020-08-14

**Authors:** Bara Singh, Siddheshwar K. Bankar, Ketan Kumar, S. S. V. Ramasastry

**Affiliations:** Organic Synthesis and Catalysis Lab, Department of Chemical Sciences, Indian Institute of Science Education and Research (IISER) Mohali Sector 81, Manauli PO, S. A. S. Nagar Punjab 140306 India ramsastry@iisermohali.ac.in ramsastrys@gmail.com

## Abstract

Correction for ‘Palladium-catalysed 5-*endo-trig* allylic (hetero)arylation’ by Bara Singh *et al.*, *Chem. Sci.*, 2020, **11**, 4948–4953, DOI: 10.1039/D0SC01932A.

After the publication of our manuscript, a reader suggested that the transformation (shown in Scheme 1) can also be described as a Nazarov-type cyclisation^1^ with palladium chloride acting as a Lewis acid. While we do not have any evidence at this stage to support the Lewis acidic behaviour of palladium chloride,^2^ however, a Nazarov-type mechanistic scenario is possible. The overall transformation can also be considered as an intramolecular ene-type reaction^3^ or as an intramolecular Friedel–Crafts-type reaction,^4^ although our data agrees better with the 5-*endo-trig* process facilitated by the LUMO umpolung.^5^ Further experimental and computational investigations are underway to elucidate the full mechanistic details.

**Scheme 1:**
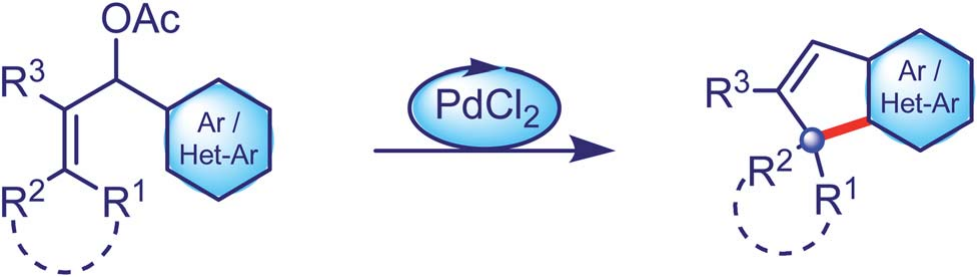
General representation of the palladium-catalysed intramolecular allylic (hetero)arylation strategy reported by our group.

We thank the reader for the thought-provoking comments and also for their interest in our work.

Previously, the methoxy (–OMe) groups in the structure **2l** (Table 2) were wrongly placed. The correct structure of **2l** is show below.
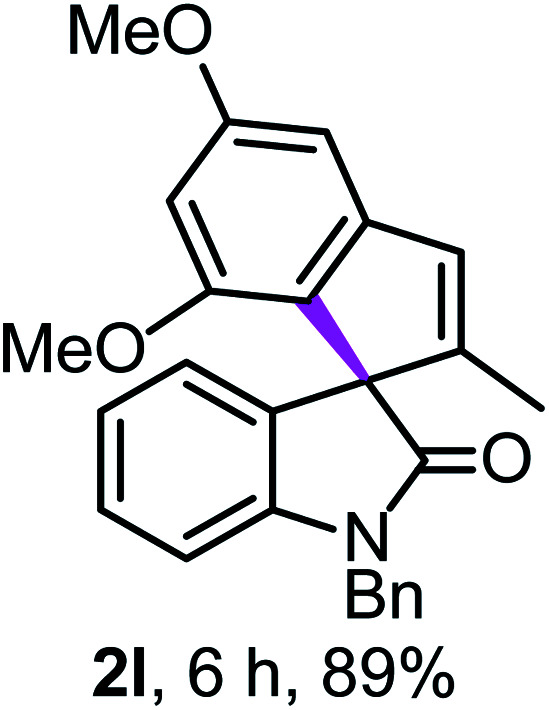


These corrections do not influence any conclusions reported in the main article.

The Royal Society of Chemistry apologises for these errors and any consequent inconvenience to authors and readers.
